# Cross-Cultural Adaptation and Validation of a Marathi Version of the Versus Arthritis Musculoskeletal Health Questionnaire (MSK-HQ)

**DOI:** 10.7759/cureus.43009

**Published:** 2023-08-05

**Authors:** Priyadarshi Prajjwal, Manasi Pimpale, Sakshi Manglik, Shraddha Nakum, Anjali Shukla, Ayush Kumar, Raunak Ranjan, Kavita Krishna, Sandeep Kansurkar

**Affiliations:** 1 Department of Clinical Immunology and Rheumatology, Bharati Vidyapeeth (Deemed To be University) Medical College, Pune, IND; 2 Physiotherapy, University of Birmingham, Birmingham, GBR

**Keywords:** marathi version, rheumatology, msk-hq, disability adjusted life years, physiotherapy, quality of life, musculoskeletal health

## Abstract

Introduction

Musculoskeletal (MSK) well-being plays a crucial role in determining one's quality of life. Musculoskeletal Health Questionnaire (MSK-HQ) score is a tool recently developed by the Versus Arthritis group of Oxford University in English to measure MSK health. Marathi is a regional language in western India spoken by more than 100 million people. There is a scarcity of valid and reliable tools to measure MSK health in this language. Hence, we decided to cross-culturally adapt and translate MSK-HQ to Marathi.

Method

We translated MSK-HQ score to Marathi (MSK-HQ-Ma) as per the International Society for Pharmacoeconomics and Outcomes Research (ISPOR) guidelines. We tested its internal consistency, construct validity and reproducibility. It was compared with other health status scores EQ-5D-5L and overall health using the Visual Analogue Scale (VAS). Test-retest reliability was tested in those subjects who were having stable MSK health after two weeks.

Results

We recruited 158 consecutive subjects attending musculoskeletal clinics who had Marathi as their native language. Mean age was 44.8±17 years, females were 78 (49%). It showed good internal consistency (Cronbach’s alpha = 0.95). For construct validity we found a strong correlation between MSK-HQ-Ma and EQ-5D-5L values (Spearman’s r = 0.82, p<0.001). There was also a good correlation between MSK-HQ-Ma and overall health by VAS (Spearman’s r = 0.76, p<0.001). An excellent test-retest reliability (Spearman’s r = 0.94, p<0.001) was seen in 105 subjects who had stable MSK condition after two weeks of first appearance.

Conclusion

The MSK-HQ-Ma instrument has demonstrated good consistency, reliability and construct validity when evaluating the musculoskeletal health of individuals who can understand the Marathi language. Hence it can be used as a validated tool for the evaluation of musculoskeletal health in western India where Marathi is a commonly used language.

## Introduction

The World Health Organization defines health as not only the absence of disease and infirmity, but also as a state of complete physical, mental, and social well-being [1]. Physical health involves the coordinated and smooth functioning of body systems, particularly the musculoskeletal (MSK) system, which is primarily composed of connective tissues such as bones, tendons, ligaments, and muscles. Injuries to these structures can lead to pain and significant disturbance in day-to-day functioning, as well as chronic disability and psychological disturbances [2,3]. Musculoskeletal diseases (MSD) have a direct influence on working capability [4] and productivity [5-7], making it necessary to assess and address musculoskeletal problems globally to improve quality of life [8].

Around 45% of adults have musculoskeletal indisposition, and this problem is particularly significant in India [9]. However, the actual burden of musculoskeletal disorders remains underestimated because some people tailor their lives to cope with these issues while others seek medical help [10].

To plan interventions and address this problem effectively, measurement is the key [11]. Unfortunately, there are currently no validated tools in the local language to measure musculoskeletal health in Indian patients. The functional scores that are currently used, such as the Health Assessment Questionnaire Disease Index (HAQ-DI) [12] and the 36-Item Short Form Health Survey questionnaire (SF-36) [13], provide limited information about MSK health. Other regional scores like the Shoulder Pain and Disability Index (SPADI) [14], Oswestry Disability Index (ODI) [15], and Western Ontario and McMaster Universities Osteoarthritis Index (WOMAC) score [16] are focused on specific areas of the MSK system, thereby underestimating their impact on social and emotional aspects.

To measure the different aspects of musculoskeletal health simultaneously, composite scores are required. The Versus Arthritis Musculoskeletal Health Questionnaire (MSK-HQ) [17] is a composite score that acknowledges these shortcomings. It was developed jointly by the Arthritis Research UK Primary Care Sciences Research Centre at Keele University and the University of Oxford and has the ability to measure impairment in physical activities and its impact on social and emotional well-being and quality of sleep [18]. The score has been validated to evaluate MSK health in musculoskeletal clinics [19] in several Asian and European languages [20-23]. These translated versions have tested for psychometric properties like internal consistency, test-retest reliability and construct validity with respect to other measures of quality of life like EQ-5D.

However, the MSK-HQ has not yet been translated into Indian languages. Therefore, we translated and cross-culturally adapted the MSK-HQ score to Marathi and compared its outcomes with the previous studies. We found that this questionnaire has the capacity to be a useful tool for objectively assessing the musculoskeletal health of Indian patients who understand Marathi.

## Materials and methods

Instrument

The MSK-HQ comprises a composite tool consisting of a Likert scale questionnaire ranging from 0 to 4 that addresses 14 domains related to musculoskeletal health. These domains encompass various aspects of physical and emotional well-being such as stiffness during the day and night, ability to walk, perform daily activities like dressing and washing, social interaction, emotional distress, understanding of MSD, and confidence to manage it. The final score is obtained by adding up the scores of each of the 14 domains. The minimum possible score is 0 while the maximum score that can be achieved is 56.

Translation

The first step in the process of cultural adaptation of the MSK-HQ questionnaire involved translating and back-translating the original questionnaire to ensure its reliability and validity. Oxford University Innovation granted us permission to translate the MSK-HQ questionnaire into Marathi, and we followed the guidelines set by the International Society for Pharmacoeconomics and Outcomes Research (ISPOR) [24] to ensure the accuracy of the translation process.

In the first phase of forward translation, two healthcare professionals who were proficient in both Marathi and English independently translated the original questionnaire. An independent investigator, who was a native Marathi speaker, then compared the two translations to identify any discrepancies. Minor differences were reconciled into a single translated version, considering the speech and writing styles commonly used by individuals, which was then reviewed by another independent investigator to eliminate any remaining discrepancies.

In the second phase, the translated version was back-translated into English by two translators who had not seen the original questionnaire. The principal investigator compared the back-translations with the original questionnaire, and most of the discrepancies were already corrected in the previous steps. The remaining minor discrepancies were modified to ensure cultural and linguistic equivalence with the original questionnaire. For complex Marathi terms, English terms were also provided in brackets for better comprehension.

**Figure 1:**
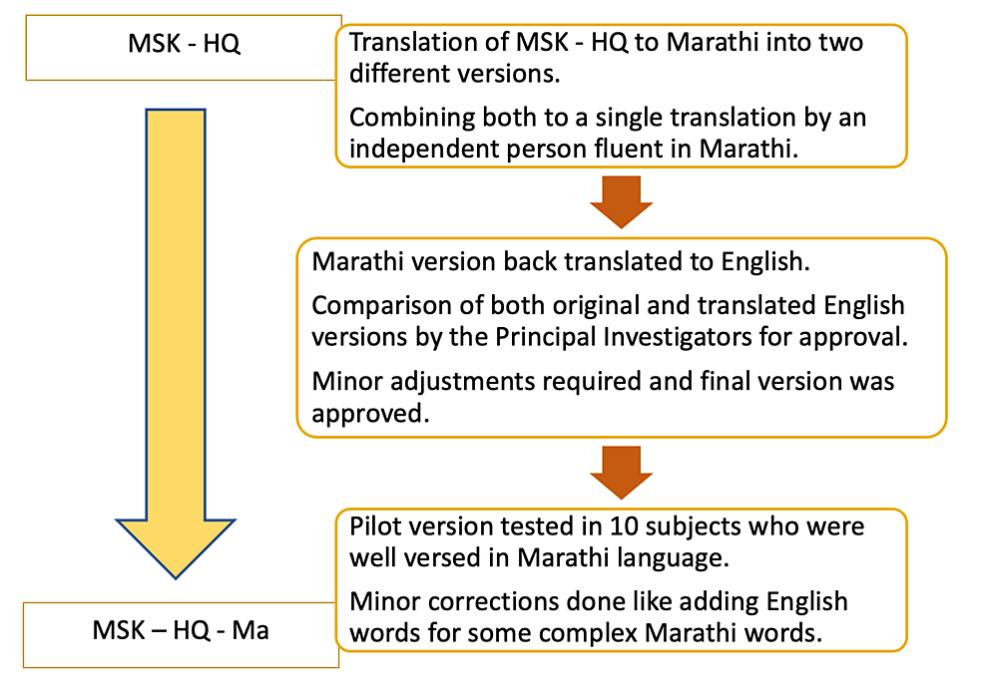
Multi-step forward and backward translations of the original English version of the Musculoskeletal Health Questionnaire (MSK-HQ) to the Marathi version (MSK-HQ-Ma)

In the third phase, a pilot test was conducted with 10 patients with musculoskeletal complaints who were attending our hospital. The patients filled out the MSK-HQ-Ma questionnaire, and the average time taken was approximately five minutes. Feedback and suggestions obtained from the patients were incorporated to make minor improvements in the questionnaire, such as increasing the font size and providing the English meaning for some of the Marathi words to aid comprehension. The final version of the Marathi questionnaire was labeled as MSK-HQ-Ma, and the translation process is depicted in Figure 1.

The study aimed to test the psychometric properties of the questionnaire on a larger group of patients and was approved by the Institutional Ethics Committee (EC/NEW/INST/2020/656).

Inclusion criteria

Consecutive patients attending the Outpatient Department of our centre with musculoskeletal complaints at the Rheumatology, Orthopaedics, Spine Surgery and Physiotherapy departments above the age of 18 years who can comfortably read and understand Marathi language were recruited.

Those with very severe pain of any aetiology, disability due to neurological causes, inability to read due to visual defects, severe hearing loss and concurrent infection were excluded.

Data collection

Each participant was given detailed and clear information about the purpose, aims and objectives of the study and was made aware that their participation is completely voluntary and confidential. Further, a written informed consent was taken from the participants. Subjects filled MSK-HQ-Ma proforma by themselves by reading it. Assistance in marking the responses was provided for subjects with a hand disability. To test for construct validity, another measure of state of health, EQ-5D-5L [25], was used in this study. Requisite permission and licence were obtained from the original publisher, EuroQOL group. The Marathi version of EQ-5D-5L [26] was used for this study which was integrated with MSK-HQ-Ma questionnaire. This was a self-reported questionnaire for which investigators provided help in understanding the question if required.

As per COSMIN (COnsensus-based Standards for the selection of health Measurement INstruments) guidelines for calculating sample size for content validation, sample size should be at least 100 [27]. The questionnaire was filled at the baseline and after two weeks for test-retest assessment while their clinical condition was found to be stable in this period. It was assumed that one-third of the subjects will not have stable MSD. To have at least 100 subjects with stable MSD, for test-retest analysis, a sample of 150 was planned. Twenty percent extra subjects were also enrolled to compensate for missing data for the first response, hence the final sample size was decided to be 180.

The responses to the MSK-HQ-Ma questionnaire were tested for internal consistency and test-retest reliability. The total MSK-HQ-Ma score was compared with the EQ-5D-5L score for testing construct validity.

Statistical analysis

The internal consistency of the measurement was examined using Cronbach’s alpha, with the acceptable alpha level being between 0.70 and 0.95. The test-retest reliability of the MSK-HQ-Ma was assessed using the non-parametric Spearman’s intraclass correlation coefficient r (ICC) with a 95% confidence interval. An acceptable level of ICC r was 0.7 or higher. Construct validity was evaluated using Spearman’s correlation analysis to determine the correlation between the MSK-HQ-Ma and the EQ-5D-5L. Same was tested for MSK-HQ-Ma against overall health assessment by Visual Analogue Scale (VAS). The level of significance was set at p≤0.05 and acceptable ICC r was 0.7 or higher.

## Results

A total of 180 subjects were enrolled. Of these 22 were excluded due to incomplete data. We analysed data for 158 subjects. In this group 78 (49%) were females and 80 were male (51%). Mean age was 44.8±17 years. Demographic details are described in Table 1.

**Table 1:** Demographic details of participants SD: Standard deviation, VAS: Visual Analogue Scale, MSK-HQ-Ma: Musculoskeletal Health Questionnaire, Marathi version

Parameter	Value
Total (n)	158
Sex	
Female	78
Male	80
Age (in years)	44.8±17
Comorbidities (n)	
Diabetes	33
Heart disease	13
Trauma	13
Joint disease	15
Brain stroke	1
Recent hospitalisation	7
Exercise- days per week (Mean±SD)	2.73±2.55
MSK-HQ-Ma (Mean±SD)	46.23±10.69
EQ-5D-5L (Mean±SD)	0.83±0.31
Overall health by VAS (Mean±SD)	82.18±19.21

Mean MSK-HQ-Ma was 46.23±10.69 among all subjects. The mean score for MSK-HQ-Ma in females and males was 42.62±11.69 and 48.38±9.26 respectively. Internal consistency as tested by Cronbach’s alpha was 0.95 which was acceptable and good. Item total score analysis is described in Table 2. Cronbach’s alpha with the missing item was almost similar for all entries in the score.

**Table 2:** Item analysis for Musculoskeletal Health Questionnaire, Marathi version (MSK-HQ-Ma)

Item No	Item	Correlation	Cronbach's Alpha with missing item	Mean with item deleted	Standard Deviation
1	Pain/stiffness during day	0.78	0.75	3.20	1.12
2	Pain/stiffness during night	0.74	0.75	3.34	1.07
3	Walking	0.73	0.75	3.25	1.07
4	Washing/dressing	0.70	0.76	3.65	0.78
5	Physical activity levels	0.74	0.75	3.39	0.90
6	Work/daily routine	0.81	0.75	3.35	1.00
7	Social activities & hobbies	0.74	0.75	3.48	0.94
8	Needing help	0.74	0.75	3.50	0.92
9	Sleep	0.71	0.75	3.38	0.96
10	Fatigue/low energy	0.66	0.75	3.07	1.07
11	Emotional well-being	0.71	0.75	3.34	1.03
12	Understanding of your condition and current treatment	0.61	0.75	3.10	1.22
13	Confidence to manage symptoms	0.62	0.75	2.69	1.51
14	Overall impact	0.87	0.75	3.16	1.09

The strongest correlation to the final score was seen with items no. 14, 6, and 1. Item 12 had the lowest correlation to the final score.

**Figure 2:**
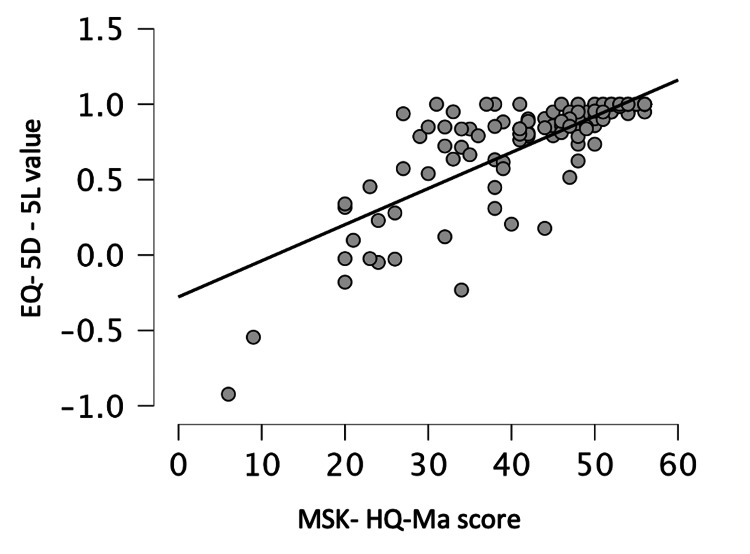
Correlation between Musculoskeletal Health Questionnaire, Marathi version (MSK-HQ-Ma) and EQ-5D-5L values (Spearman’s interclass coefficient Rho = 0.82)

**Figure 3:**
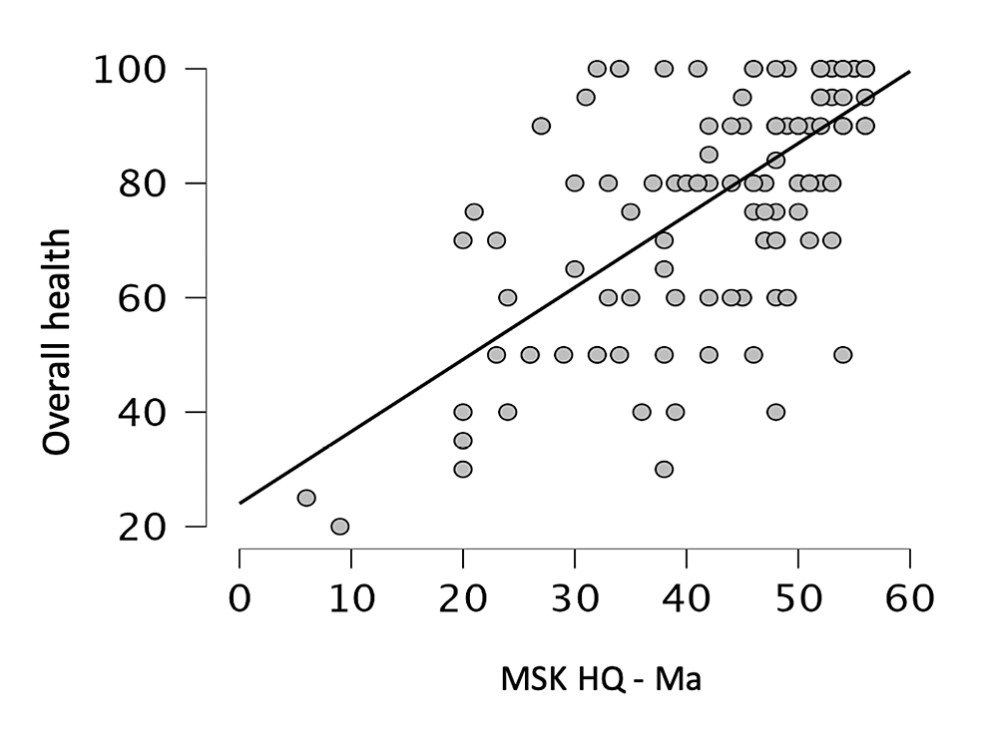
Correlation between Musculoskeletal Health Questionnaire, Marathi version (MSK-HQ-Ma) and overall health by Visual Analogue Scale (Spearman’s interclass coefficient Rho = 0.76)

Construct validity was evaluated by testing the correlation between MSK-HQ-Ma and EQ-5D-5L values. Good correlation was observed between these scores (Spearman’s r=0.82, p<0.001) (Figure 2). There was also good correlation between MSK-HQ-Ma and overall health by VAS (Spearman’s r=0.76, p<0.001) (Figure 3).

These findings suggested a good match of MSK-HQ-Ma with other measures of state of health.

There was a significant negative correlation of MSK-HQ-Ma to the age of the participants (Spearman’s r=-0.4, p<0.001) which was expected. A significant correlation was seen in number of days of exercise per week and MSK-HQ-Ma (Spearman’s r=0.3, p<0.001) suggesting more regular exercise was associated with better MSK health.

**Figure 4:**
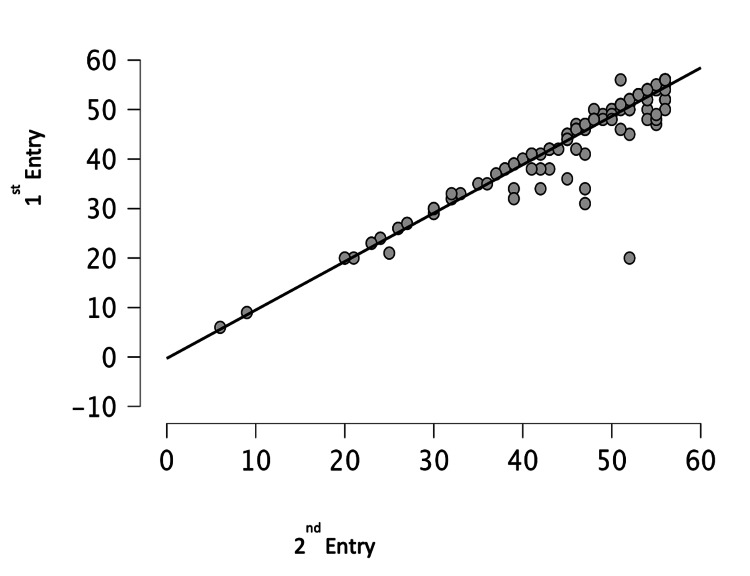
Correlation between test-retest versions of Musculoskeletal Health Questionnaire, Marathi version (MSK-HQ-Ma) (Spearman’s interclass coefficient Rho = 0.95, p<0.005)

Among all the recruited subjects for the first entry, 146 could be contacted after two weeks for recall testing. The health condition of 105 of them was reported to be stable and similar to the first entry. Test-retest reliability was tested by repeat questionnaire in them and a strong correlation was observed between the two readings (Spearman’s r=0.95, p<0.005) (Figure 4).

## Discussion

The objective of this study was to translate and adapt the MSK-HQ score into Marathi and evaluate its psychometric properties. The translated version demonstrated good internal consistency, reliability, and fair construct validity, as evidenced by its good correlation with EQ-5D-5L score and overall health measured by VAS. Moreover, the study found a negative correlation with age and a positive correlation with exercise, which strengthens the outcome.

MSK-HQ has been previously translated into several Asian and European languages, and our study showed a similar level of reliability with the original English version and slightly better than the Arabic and Italian versions. The correlation of MSK-HQ-Ma with EQ-5D-5L was 0.82. It was better than previously translated versions like the Arabic version (MSK-HQ-Ar; 0.71) [20] and the Italian version (MSK-HQ-I; 0.67) [23]. In item analysis, the strongest correlation to the final score was seen with item 14 and item 6 which dealt with interference in work or daily routine and overall impact (r=0.87 and r=0.81). In the original study, item 6 exhibited the strongest correlation. The weakest correlation was seen in item 12 (r=0.61) [25] which refers to understanding of current condition and treatment. It was similar to the original study which also recorded it to have the lowest correlation (ICC=0.41). In another study where MSK-HQ was tested for patients with arthritis [28], Cronbach’s alpha was 0.93, which is equivalent to that of our study.

This study also demonstrated excellent test-retest reliability. Strong correlation (Spearman’s r=0.95) observed between two readings taken two weeks apart in those with stable disease underlines good reproducibility and stability of its results. This was similar to other translated versions like the Arabic version (MSK-HQ-Ar; r=0.94) [20] and the Italian version (MSK-HQ-I; r=0.96) [23].

For construct validity we compared MSK-HQ-Ma with EQ-5D-5L score value. This study utilised recently published value set of EQ-5D-5L for Indian population [29]. There was a strong correlation with EQ-5D-5L value (r=0.82, p<0.005). This was stronger than was seen with other translated versions like Arabic MSK-HQ-Ar (r=0.72) [20] and similar to the original English MSK-HQ (r=0.82) [25]. Overall mean of MSK-HQ score in this study was 46.23 which was higher than with other versions like the original English MSK-HQ (28.62), Arabic MSK-HQ-Ar (32.29) and Italian MSK-HQ-I (37.39). This may be because our cohort had subjects from a better spectrum of MSK health.

The results of this study validate the utility of the Marathi version of MSK-HQ to assess musculoskeletal health of the people who can read and understand Marathi. This can be a valuable instrument as there are no other validated tools available in this language to assess MSK health. This can help authorities and researchers to plan interventions to improve quality of life, reduce burden of MSDs in society and reduce the economic outfall.

The study's limited focus on a particular geographic region or healthcare setting might reduce the generalisability of its findings. Moreover, there could be a risk of selection bias stemming from the recruitment process used to identify participants. If certain healthcare facilities were targeted for recruitment, it is possible that they do not represent the full spectrum of patients with musculoskeletal conditions. Additionally, the study may have overlooked some confounding variables that could affect the results, such as coexisting treatments, co-morbidities, or lifestyle factors. These factors could have an impact on the outcome measures but were not necessarily accounted for in a thorough manner.

## Conclusions

The Marathi version of MSK-HQ, MSK-HQ-Ma, has exhibited sound psychometric properties, including reliability, reproducibility, and strong construct validity. It can be used as a valid tool for measuring musculoskeletal health in patients who are able to read and comprehend Marathi. It is a convenient instrument in clinical settings and can be applied to monitor the progression of musculoskeletal diseases. This will fulfill the necessary requirement of a validated instrument in the local language for the subjects from Marathi-speaking regions.
